# Osteogenic Property Regulation of Stem Cells by a Hydroxyapatite 3D-Hybrid Scaffold With Cancellous Bone Structure

**DOI:** 10.3389/fchem.2021.798299

**Published:** 2021-11-19

**Authors:** He Xia, Lun Dong, Min Hao, Yuan Wei, Jiazhi Duan, Xin Chen, Liyang Yu, Haijun Li, Yuanhua Sang, Hong Liu

**Affiliations:** ^1^ State Key Laboratory of Crystal Materials, Shandong University, Jinan, China; ^2^ Department of Breast Surgery, Qilu Hospital, Shandong University, Jinan, China; ^3^ Department of Obstetrics and Gynecology, Qilu Hospital, Shandong University, Jinan, China; ^4^ Key Laboratory of Cardiovascular Proteomics of Shandong Province, Department of Geriatric Medicine, Qilu Hospital, Shandong University, Jinan, China

**Keywords:** bone tissue engineering, hydroxyapatite, 3D scaffold, hADSCs, osteogenesis differentiation

## Abstract

Cancellous bone plays an indispensable role in the skeletal system due to its various functions and high porosity. In this work, chitosan and hydroxyapatite nanowires (CS@HAP NWs) hybrid nanostructured scaffolds with suitable mechanical properties, high porosity and a fine porous structure were prepared to simulate the 3-dimensional structure of cancellous bone. The 3D-hybrid scaffolds promote cell adhesion and the migration of human adipose-derived stem cells (hADSCs) inside the scaffolds. The cavities in the scaffolds provide space for the hADSCs proliferation and differentiation. Moreover, the various contents of HAP and the induced mechanical property changes regulate the differentiation of hADSCs toward osteoblasts. Overall, cellular fate regulation of hADSCs via rationally engineered HAP-based hybrid scaffolds is a facile and effective approach for bone tissue engineering.

## Introduction

Bone defects caused by trauma, congenital anomalies and tumor resection create a huge demand for bone grafts (Zhang et al., 2019; Koons et al., 2020; Collins et al., 2021). Despite the inherent self-repair ability of bone, the gold standard for defect treatment is still autologous bone transplantation (Zhang et al., 2017; Annamalai et al., 2019; Li et al., 2020). With the progression of tissue engineering, biomimetic bone scaffolds can guide tissue regeneration with assembled stem cells (Zhao et al., 2019; Swanson et al., 2021; You et al., 2021). Therefore, they have considered a cost-effective and osteoinductive strategy to replace autologous bone grafts (Cui et al., 2019).

As previously reported, the physical and biochemical properties of the matrix regulate the fate of adherent cells (Kim et al., 2017; Teotia et al., 2017; Swanson et al., 2021). There are many studies concerning the modification of the matrix to provide a suitable bioenvironment for the osteogenic property regulation of stem cells (Jo et al., 2017; Yang et al., 2018; Wang et al., 2019; Zhong et al., 2021). Researchers have come to a consensus on the fundamental requirements of biomimetic bone scaffolds (Chen et al., 2016; Lin et al., 2019). First, great biocompatibility and suitable biochemical properties can facilitate the survival of stem cells (Ma et al., 2017; Patil et al., 2017). Second, the appropriate physical properties provide an environment for cell proliferation and differentiation, such as a highly interconnected structure, porosity and pore size, as well as adequate mechanical properties (Gaihre and Jayasuriya, 2018; Jiang et al., 2019; Wang et al., 2021). Third, the biomimetic and osteoconductive components provide a suitable extracellular environment for osteogenic progression (Liu et al., 2016; Chen et al., 2017; Cheng et al., 2020; Lee et al., 2020; Brennan et al., 2021; Sun et al., 2022). Cancellous bone is a typical example and it is comprised of hydroxyapatite (HAP, Ca_10_(PO_4_)_6_(OH)_2_) crystals deposited within an organic matrix (Oftadeh et al., 2015; Zhang et al., 2018; Wittig et al., 2019). It has high porosity and an interconnected porous structure that can serve as an incubator for cell in-growth and bone regeneration (Wang et al., 2019).

Inspired by the structure of cancellous bone, by replacing collagen with chitosan (CS) as the organic matrix, CS@HAP nanowires (CS@HAP NWs) aerogel scaffolds with highly interconnected porous structures were synthesized via a freeze-drying process. Considering the basic characteristics of bone scaffolds, the physical properties and chemical properties of the scaffolds were tailored. hADSCs were cultured on aerogels to investigate the influence of various 3D scaffolds on cellular activities, such as cell adhesion, migration and proliferation. In addition, the osteogenic properties of aerogel scaffolds indicated the synergy and coordination of HAP NWs and the porosity in the regulation of osteogenic differentiation. This study not only provides a simple method to prepare a biomimetic fine-tuned porous scaffold for bone grafts but also highlights potential applications of scaffolds in improving their therapeutic efficacy for regenerative medicine.

## Materials and Methods

### Chemicals

For material synthesis, sodium oleate (C_18_H_33_NaO_2_, chemical pure), calcium chloride (CaCl_2_, 96.0%), sodium dihydrogen phosphate (NaH_2_PO_4_, 99.0%), hexane (C_6_H_14_, 97.0%), ethanol (C_2_H_6_O, 99.7%), acetic acid (C_2_H_4_O_2_, 99.5%) and sodium hydroxide (NaOH, 96.0%) were purchased from Sinopharm Chemical Reagent Co., Ltd. Chitosan (C_6n_H_11n_NO_4n_, deacetylation degree ≥95%) and genipin (C_11_H_14_O_5_, 98%) were purchased from Shanghai Macklin Biochemical Co., Ltd. All of the chemicals were used without further purification.

For the cell experimental process, the α−minimum essential medium (α−MEM), Dulbecco’s modified Eagle’s medium (DMEM), fetal bovine serum (FBS) and penicillin/streptomycin were purchased from Gibco (America). Calcein acetoxymethyl ester (calcium AM) and propidium iodide (PI) were purchased from Beijing Solarbio Science and Technology Co., Ltd. Cell counting kit-8 (CCK-8) was purchased from Dojindo (Japan). FITC-phalloidin was purchased from Yeasen Biotechnology (Shanghai) Co., Ltd. 4′,6-Diamidino-2-phenylindole (DAPI) was purchased from Abcam (United Kingdom). Alizarin Red S was purchased from Shanghai Yuanye Bio-Technology Co., Ltd. (China).

### Synthesis of HAP Nanowires, CS Aerogel and CS@HAP NWs Aerogel

Hydroxyapatite (HAP) nanowires were prepared by the hydrothermal method. Briefly, sodium oleate (2.40 g) was dissolved in a solution that contained 25 ml of deionized water and 5 ml of ethanol under vigorous stirring. CaCl_2_ (0.22 g) was dissolved in 20 ml of deionized water and then added to the sodium oleate solution. After 1 h of stirring, NaH_2_PO_4_ (0.28 g) was dissolved in 25 ml of deionized water and dropped into the above solution, and a white suspension formed. It was moved to a Teflon lined autoclave and heated at 200°C for 40 h. After 40 h, HAP nanowires were obtained after thorough washing with hexane, ethanol and water.

To prepare the CS@HAP NWs aerogel, chitosan (0.40 g) was dissolved in 2% acetic acid solution (20 ml) and stirred for 2 h. Then, a certain amount of HAP nanowires was added to the CS solution and stirred for 30 min. The chemical crosslinking reagent genipin was added to the solution and stirred for 12 h. The mass ratio of CS to genipin was 400 to 1. Then, the solution was poured into the cylinder mold and freeze-dried for 48 h into an aerogel. The obtained aerogel was immersed in 1 M NaOH solution for 24 h to remove the residual acid. Finally, it was thoroughly washed with deionized water to remove the NaOH.

### Characterization

The morphology of the HAP nanowires, CS aerogel and CS@HAP NWs aerogel was observed by S-4800 scanning electron microscopy (SEM, Hitachi, Japan). X-ray diffraction (XRD) was recorded on a powder diffractometer equipped with a Cu Kα sealed tube (Bruker D8 advance, Germany) to analyze the compositions. Fourier transform infrared spectroscopy (FTIR) was used to qualitatively analyze the ion groups of HAP nanowires, CS aerogel and CS@HAP NWs aerogel (Nicolet Nexus 670, Thermo Fisher Scientific, Inc.). The porosity of the scaffold was measured by the liquid displacement method. Briefly, at room temperature, the aerogel was dipped into anhydrous ethanol (V_1_), and the new volume (V_2_) was recorded. After saturated adsorption, the aerogel was removed, and the volume of ethanol (V_3_) was recorded. The porosity was calculated with the following formula:

$$Porosity\;\left(\%\right)=\left(V_{1}-V_{3}\right)\times 100/\left(V_{2}-V_{3}\right)$$

The compression stress-strain curves of the CS aerogel and the CS@HAP NWs aerogels were measured by a texture analyzer (TA. XTplusC, Stable Micro Systems). Young’s modulus was calculated by the slope of a straight line fitted for the stain-stress curves from 5 to 15% strain. To study the structural collapse of the CS aerogel modified with various concentrations of HAP nanowires, the CS aerogel and CS@HAP NWs aerogels were immersed in PBS for 2 weeks. Then, the exfoliated debris was collected by centrifugation at 10,000 rpm, and florescence images were captured by a fluorescence inverted microscope.

### Cell Culture

Human adipose-derived stem cells (hADSCs) were obtained from the Qilu Hospital of Shandong University in Jinan, and their use met the requirements of ethics and morality. The hADSCs were cultured in α-MEM supplemented with 10% FBS and 1% penicillin/streptomycin, incubated in a 5% CO_2_ atmosphere at 37°C, and the growth medium was replaced every 2 days. The cells from passages 3–5 were used in this study. For osteogenesis differentiation, 1 × 10^–3^ M dexamethasone, 10 × 10^–3^ M ascorbic acid, and 1 M β-glycerophosphate, serving as nutrient factors, were added to the basic growth medium.

### Live and Dead Staining

Live and dead staining was carried out according to the manufacturer’s instructions. For 2-dimensional (2D) culturing, 0.1 ml of the cell suspension containing 1 × 10^4^ cells was seeded on 24-well plates, and 0.4 ml of the growth medium was added. Then, the aerogels were immersed in the medium to simulate the growth environment of hADSCs cultured on the surface of the aerogel. For 3-dimensional (3D) culturing, 0.1 ml of the cell suspension containing 2 × 10^4^ cells was seeded on the aerogels in 24-well plates and incubated at 37°C for 30 min. Then, 0.5 ml of growth medium was added. After culturing the hADSCs in growth medium in 2D and 3D environments for 7 days or 14 days, 500 μl of growth medium containing 1 M calcein AM and 6 M PI was added to the 24-well plates. After incubating for 30 min at 37°C, the cells were washed three times with PBS and observed by fluorescence inverted microscopy or confocal laser scanning microscopy (CLSM).

### Cytoskeleton and Nuclei Staining

Cytoskeletal and nuclear staining was carried out according to the manufacturer’s instructions. Briefly, 0.1 ml of the cell suspension containing 2 × 10^4^ cells was seeded on the aerogels in 24-well plates and incubated at 37°C for 30 min. Then, 0.5 ml of the growth medium was added. After culturing the hADSCs in growth medium in 2D and 3D environments for 7 days, the aerogel containing hADSCs was washed with PBS three times. Then, the samples were fixed with a 4% paraformaldehyde solution for 20 min and washed with PBS three times. After fixing, the samples were permeabilized for 10 min with 0.1% Triton X-100 and 5% bovine serum albumin (BSA) solution for 30 min at room temperature, followed by incubation with phalloidin-conjugated Alexa Fluor 568 for 30 min to stain the cytoskeleton and 4′,6-diamidino-2-phenylindole (DAPI) for 3 min to stain the nuclei. After washing with PBS three times, fluorescence images of the stained samples were captured by CLSM.

### Real-Time Quantitative Polymerase Chain Reaction

The hADSCs were cultured on the aerogels in differentiation medium for 7 and 14 days. Total RNA was extracted from the cells by TRIzol reagent (Thermo Fisher Scientific, Waltham, MA, United States) according to the manufacturer’s instructions. The concentration and purity of the RNA were determined by a Q-5000 spectrophotometer (Quawell, Q-5000, America) at 230, 260, and 280 nm. Finally, the gene expression levels of RUNX2, OPN, OCN, BMP2, CD44, and OCT4 were measured by a 7500 Real-Time PCR system (Applied Biosystems, Germany). The forward and reverse primers used in this study are listed in Supplementary Table S1. The target gene expression was normalized to that of β-actin and expressed as the mean ± standard deviation.

### Immunofluorescence Staining

The hADSCs were cultured on the surface of the aerogels at a density of 1 × 10^4^ cells per well in differentiation medium for 21 days. After washing with PBS 3 times, the cells were fixed with 4% paraformaldehyde for 20 min, permeabilized with 0.1% Triton X-100 for 10 min and blocked with 5% BSA for 30 min at room temperature. Then, the cells were incubated overnight at 4°C with primary antibodies against OPN (mouse monoclonal anti-OPN, Abcam, United Kingdom) and against OCN (rabbit polyclonal anti-OCN, Abcam, United Kingdom) in 1% bovine serum albumin. Then, the cells were incubated with the secondary antibody labeled with Alexa Fluor 594 for 1 h at room temperature. After washing three times with PBS, the cells were stained with phalloidin-conjugated Alexa Fluor 488 for 30 min and DAPI for 5 min. Finally, fluorescence images were captured by CLSM.

### Alizarin Red S Staining

hADSCs were cultured on the surface of the aerogels at a density of 1×10^4^ cells per well in differentiation medium for 21 days for Alizarin Red S staining. The cells cultured for 21 days were washed with PBS 3 times and fixed with 4% paraformaldehyde solution for 20 min at room temperature. Then, the cells were stained with Alizarin Red 2% (w/v) for 10 min. After washing with deionized water 3 times, the samples were observed by a digital camera.

## Results and Discussion

### Preparation and Characterization of the CS@HAP NWs Aerogel

**FIGURE 1:**
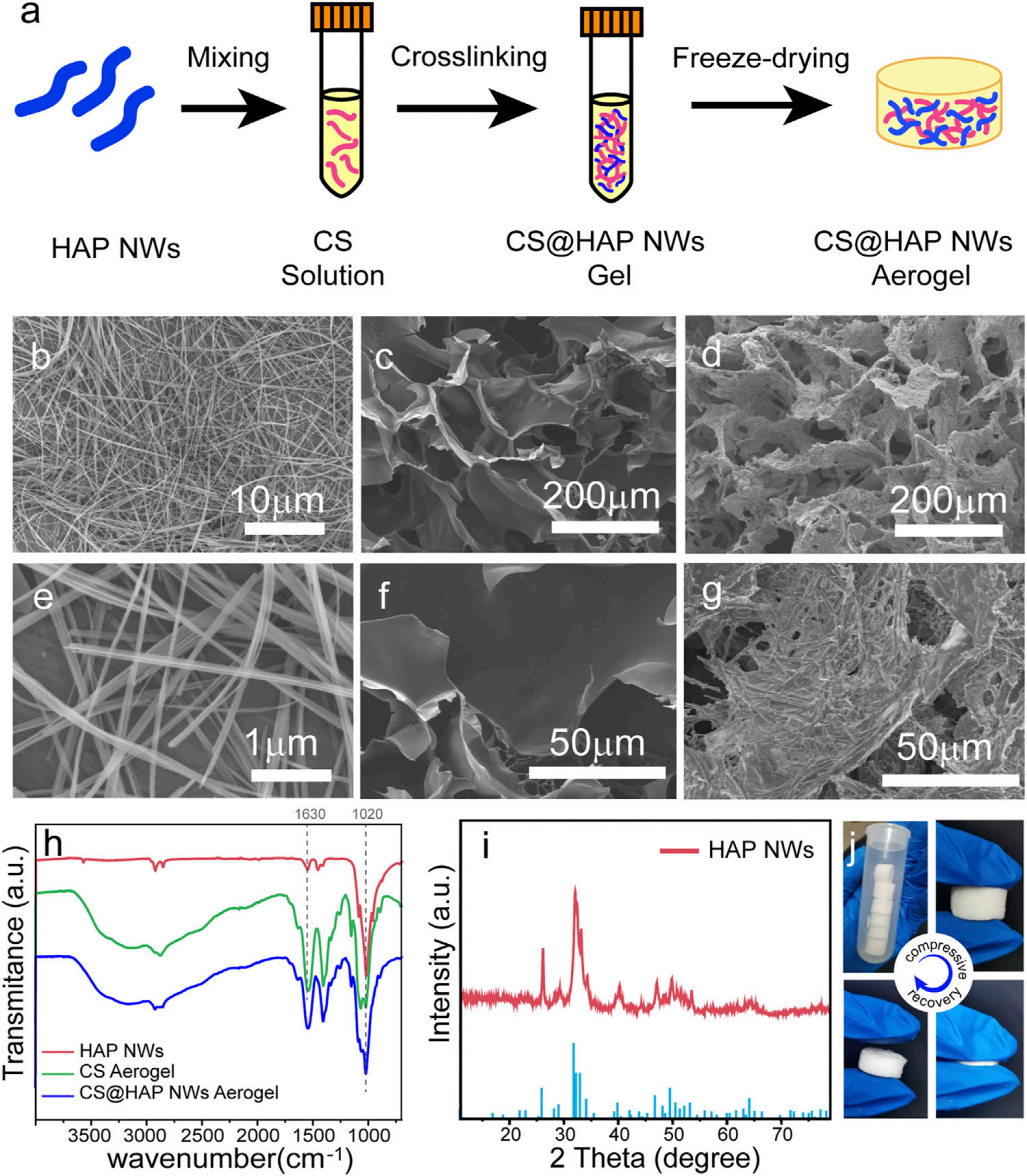
**(A)** Illustration of the preparation process of the CS@HAP NWs aerogel. **(B,E)** SEM images of the HAP nanowires, **(C,F)** CS aerogel and **(D,G)** CS@HAP NWs aerogel. **(H)** FTIR images of the HAP nanowires, CS aerogel and CS@HAP NWs aerogel. **(I)** XRD pattern of the HAP nanowires. **(J)** Digital images of the CS@HAP NWs aerogel and the recovery ability of the aerogel after applying a compressive load.

The preparation process of the CS@HAP NWs aerogel is illustrated in Figure 1a. Briefly, HAP NWs were synthesized by the hydrothermal method. After mixing with CS solution to form a homogeneous hybrid gel, a naturally low-toxicity cross-linking agent, genipin, was used to crosslink the primary amino groups on the chitosan and form a cross-linked hybrid gel. A porous CS@HAP NWs aerogel was obtained after the freeze-drying process.

The SEM images of the HAP NWs, CS aerogel, and CS@HAP NWs aerogel are shown in Figures 1B–G. The HAP nanowires are highly uniform with lengths over 20 μm and widths below 100 nm. Self-assembled nanowire bundles should form during the thorough removal of the oleate groups from the surface of the HAP NWs. (Figures 1B,E). Both the pure CS aerogel (Figures 1C,F) and CS@HAP NWs aerogel (Figures 1D,G) prepared by the freeze-drying method possess fine porous structures. The micrometer cavities provide the incubation space for the cells. The interconnections will support mass transport and cell migration (Jiang et al., 2019). The wall of the CS aerogel is thin, and the surface is smooth, while the wall of the CS@HAP NWs aerogel is thick, and the surface is rough due to the existence of HAP NWs.

**FIGURE 2:**
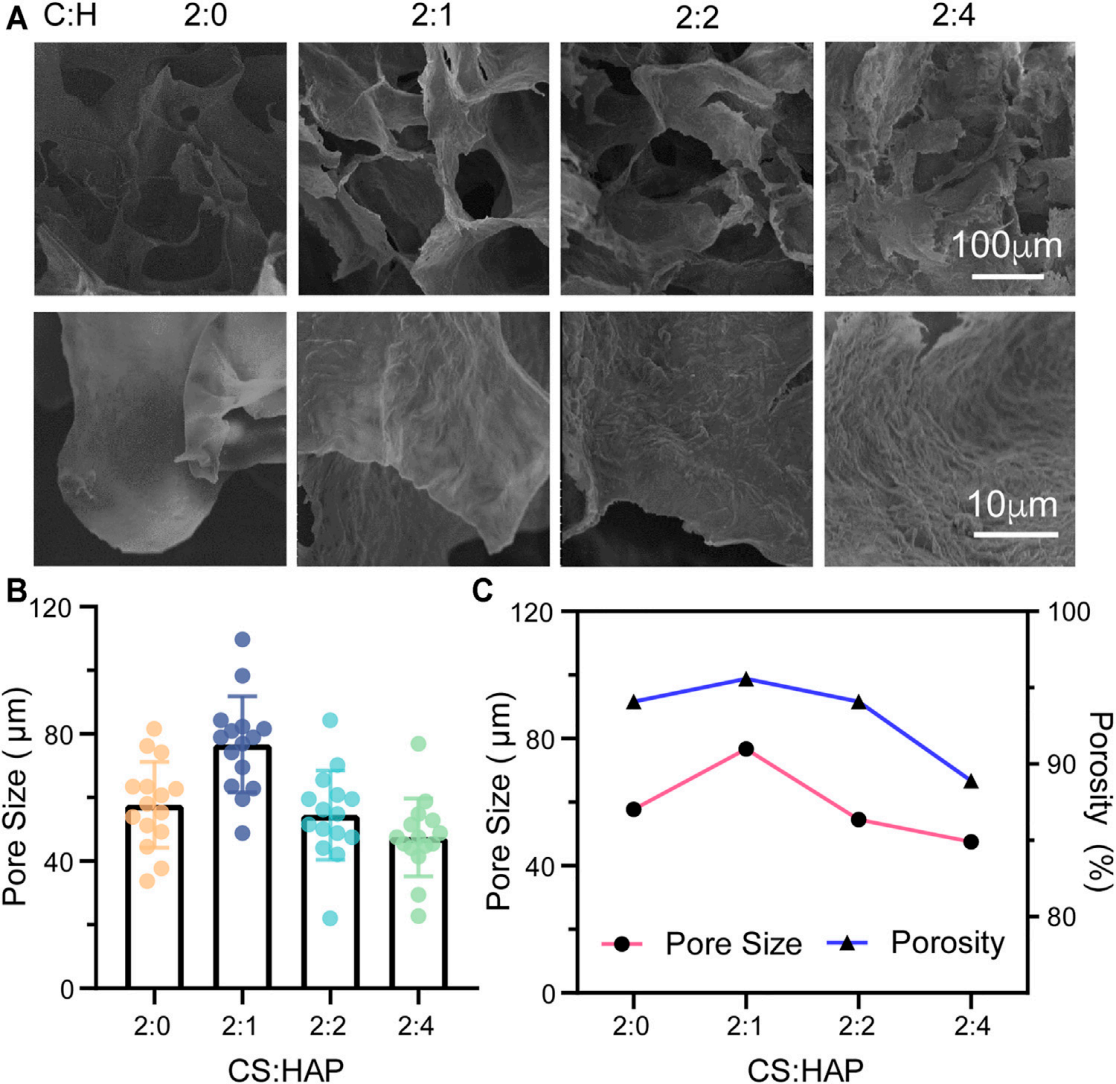
**(A)** Morphology of the CS aerogels containing various concentrations of HAP nanowires. **(B)** Pore size of the CS aerogel containing various concentrations of HAP nanowires. The pore size was measured by ImageJ from the SEM images (*n* = 15). **(C)** Porosity and pore size of the CS aerogel containing various concentrations of HAP nanowires.

Figure 2H shows the FTIR spectra of the HAP NWs, CS aerogel and CS@HAP NWs aerogel. The absorption peak at 1020 cm^−1^ in the FTIR spectra of the HAP NWs and CS@HAP NWs aerogel is attributed to the asymmetrical P-O stretching vibration of the phosphate group, which is a typical peak of HAP NWs(Zhang et al., 2018; Zhu et al., 2019). The absorption peaks at 1630 cm^−1^ of the CS aerogel and CS@HAP NWs aerogel are attributed to the C=O stretching vibration of the amide in CS. The X-ray diffraction (XRD) pattern of the HAP NWs shown in Figure 1I can be assigned to the standard HAP phase (standard PDF card: 74-0566) (Hao et al., 2021). As shown in Figure 1J, the CS@HAP NWs aerogel has excellent elastic properties and it can recover its original shape after removing the compressive load.

The physical properties of bioscaffolds, such as their morphology, porosity and mechanical properties, have a crucial influence on cell activities (Collins et al., 2021). Based on the specific structure of cancellous bone, a porous structure is required (Oftadeh et al., 2015). Therefore, the porous structures of CS@HAP NWs aerogels with various concentrations of HAP nanowires were considered according to their SEM images and porosity analysis (Figure 2). As shown in Figure 2A, the increase in the content of the HAP NWs does not destroy the interconnected porous structure. The roughness of the walls increases in the aerogels with a higher HAP NWs content. Surface roughness is also crucial for cell-scaffold interactions and osteogenesis differentiation (Levengood and Zhang, 2014). Therefore, roughness regulation by HAP NWs would benefit the osteogenic properties of the scaffold.

The pore sizes were analyzed by ImageJ according to the SEM images. As illustrated in Figure 2B, the mean pore sizes are 57.7, 76.7, 54.5, and 47.5 μm for the aerogel with various mass ratios of CS to HAP NWs of 2:0, 2:1, 2:2, and 2:4, respectively. The porosities of the aerogels are 94.1, 95.6, 94.1, and 88.9% with various mass ratios of CS to HAP NWs of 2:0, 2:1, 2:2, and 2:4, respectively, (Figure 2C). It should be noted that the aerogel (C:H = 2:1) possesses the highest porosity with the largest average pore size. The porosity and pore size of the aerogels decrease with an increasing HAP NWs content. Cancellous bone requires a high porosity of over 70% (Herford et al., 2014). This implies that the manufactured scaffold should possess a higher porosity. Both the CS aerogel and CS@HAP NWs aerogels were prepared with high porosity while C:H = 2:1 aerogel possesses the highest porosity. Thus, the cell viability and osteogenic process in the aerogel with proper contents of HAP may be improved.

**FIGURE 3:**
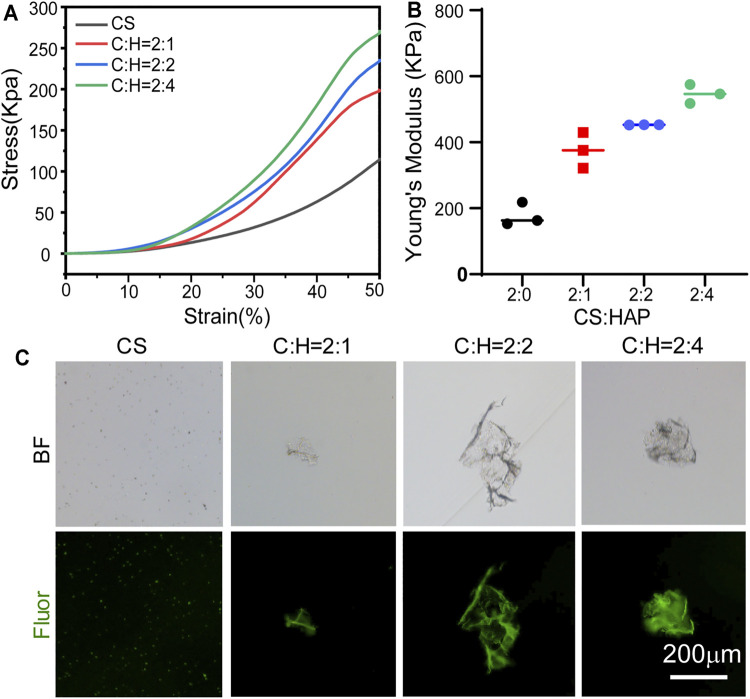
Mechanical properties of the CS@HAP NWs aerogel. **(A)** Young’s modulus of the CS aerogel containing various concentrations of HAP nanowires. **(B)** Stress-strain curve of the CS aerogel containing various concentrations of HAP nanowires. **(C)** Structure collapse properties of the CS aerogel containing various concentrations of HAP nanowires.

The mechanical properties of the aerogels were studied (Figure 3). The compression stress-strain curves of the CS aerogel and CS@HAP NWs aerogel are shown in Figure 3A. The curves show the typical behavior of elastic materials, which is consistent with the recovery ability of the aerogel after applying compression (Figure 1J). The contents of HAP NWs influence the stiffness of the aerogel. As shown in Figure 3B, the Young’s modulus of the CS@HAP NWs aerogel increased with the incorporation of HAP NWs. The Young’s modulus of the pure CS group (C:H = 2:0) was approximately 218 kPa, while the Young’s modulus of the C:H = 2:1, C:H = 2:2, and C:H = 2:4 aerogel groups reached 375, 452, and 547 kPa, respectively. Thus, the mechanical properties were improved with the addition of HAP NWs, and the C:H = 2:4 aerogel group possessed the highest Young’s modulus and best mechanical property.

It has been reported that scaffold porosity and mechanical properties are intimately related (Levengood and Zhang, 2014). As mentioned above, the C:H = 2:4 aerogel group with a higher HAP NWs content correlates with smaller pores and lower porosity (Figure 2C). Figures 3A,B indicates that a higher content of HAP NWs led to an improved mechanical strength, which further proves the intimate relationship among the porosity, pore size and mechanical properties. In particular, the compressive strength of scaffolds with the same chemical composition tends to increase with smaller pore size, lower porosity, and thicker pore walls.

In addition, the structural stability of the CS aerogel with various contents of HAP nanowires was investigated, and the structural collapse was recorded by the formation of debris (Figure 3C). After 2 weeks of immersion, part of the self-fluorescent cross-linked chitosan fell off the aerogel. In the pure CS aerogel group, a large number of small particles were observed, while some small pieces of bulk CS@HAP NWs were observed in the CS@HAP NWs aerogel group. This indicates that the structure collapse is due to degradation of the cross-linked chitosan.

### Cell Viability and Spreading Morphologies of hADSCs Cultured on the CS@HAP NWs Aerogels

**FIGURE 4:**
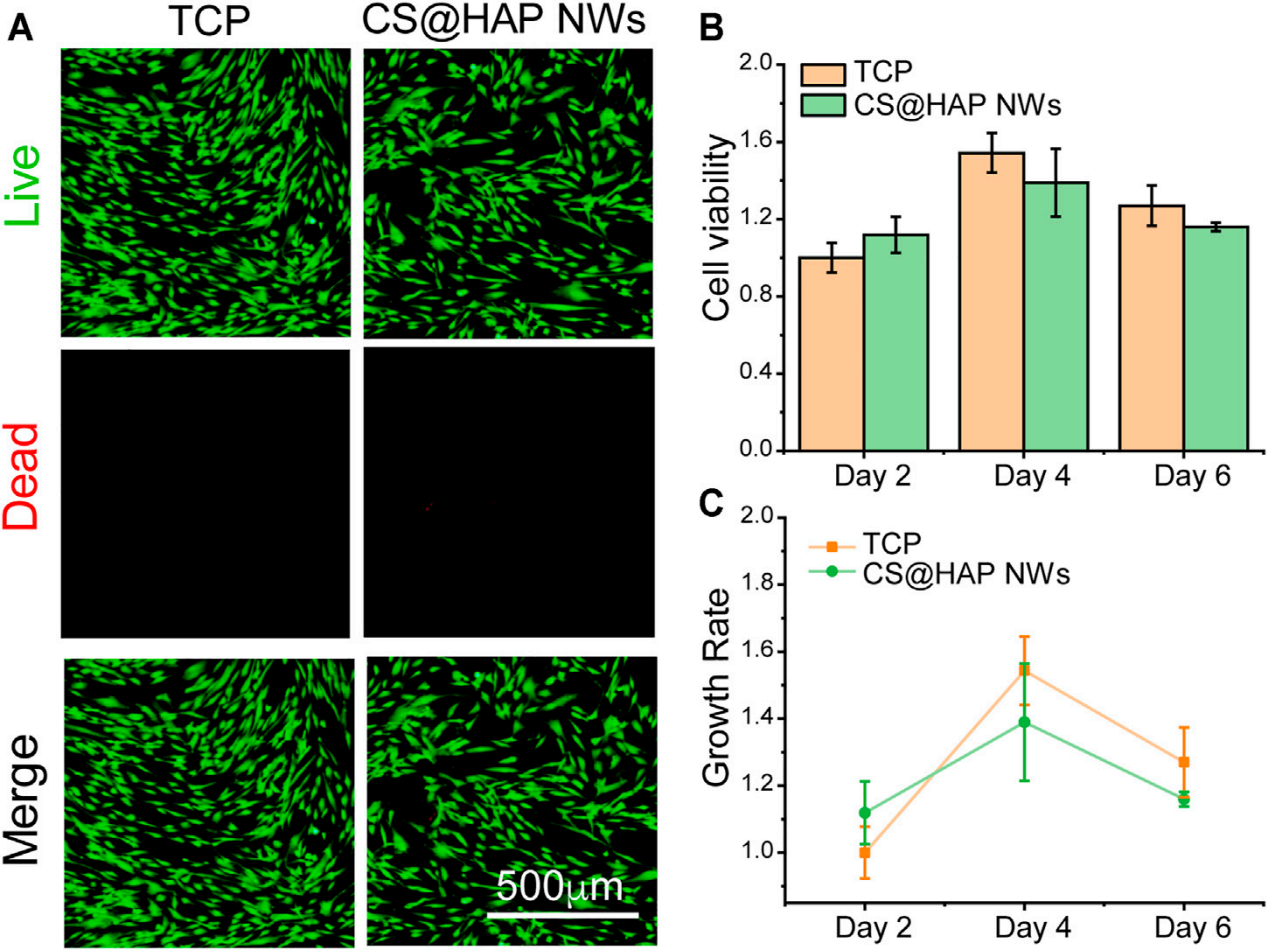
Cell viabilities of hADSCs cultured with 2D HAP nanowires. **(A)** Live and dead staining of hADSCs cultured with 0 and 100 μg/ml HAP nanowires for 3 days in growth medium. **(B–C)** CCK-8 analysis and growth rate of hADSCs cultured with 0 and 100 μg/ml HAP nanowires for 3 days in growth medium.

Good biocompatibility of scaffolds for bone tissue engineering is essential for cell activities, encouraging good attachment with proper cell growth and differentiation. The biocompatibility of the scaffold is related to both the chemical composition and the hierarchical structure. To investigate the biocompatibility of chitosan and hydroxyapatite nanowires (CS@HAP NWs) aerogel, human adipose-derived stem cells (hADSCs) were seeded on 24-well culture plates, and chitosan-coated hydroxyapatite nanowires (CS@HAP NWs) were added to the medium to explore the cellular toxicity of the chemical composition. Live and Dead Staining and Cell Counting Kit-8 (CCK-8) assays were carried out to qualitatively and quantitatively evaluate the viability of the hADSCs, respectively. As shown in Figure 4A, few dead cells stained red by propidium iodide (PI) were detected in the cells cultured with 100 μg/ml CS@HAP NWs for 3 days, indicating the good biocompatibility of the CS@HAP NWs. As shown in Figure 4B, the cell viability of the hADSCs cultured with 100 μg/ml CS@HAP NWs changed little after 48 h of culture compared with the control group. After culturing for 96 h, the cell viability of the hADSCs cultured with CS@HAP NWs increased by ∼27% which is slightly lower than that of the control. It should be assigned to the improved osteogenesis differentiation tendency of hADSCs culturing with CS@HAP NWs compared to the control.

**FIGURE 5:**
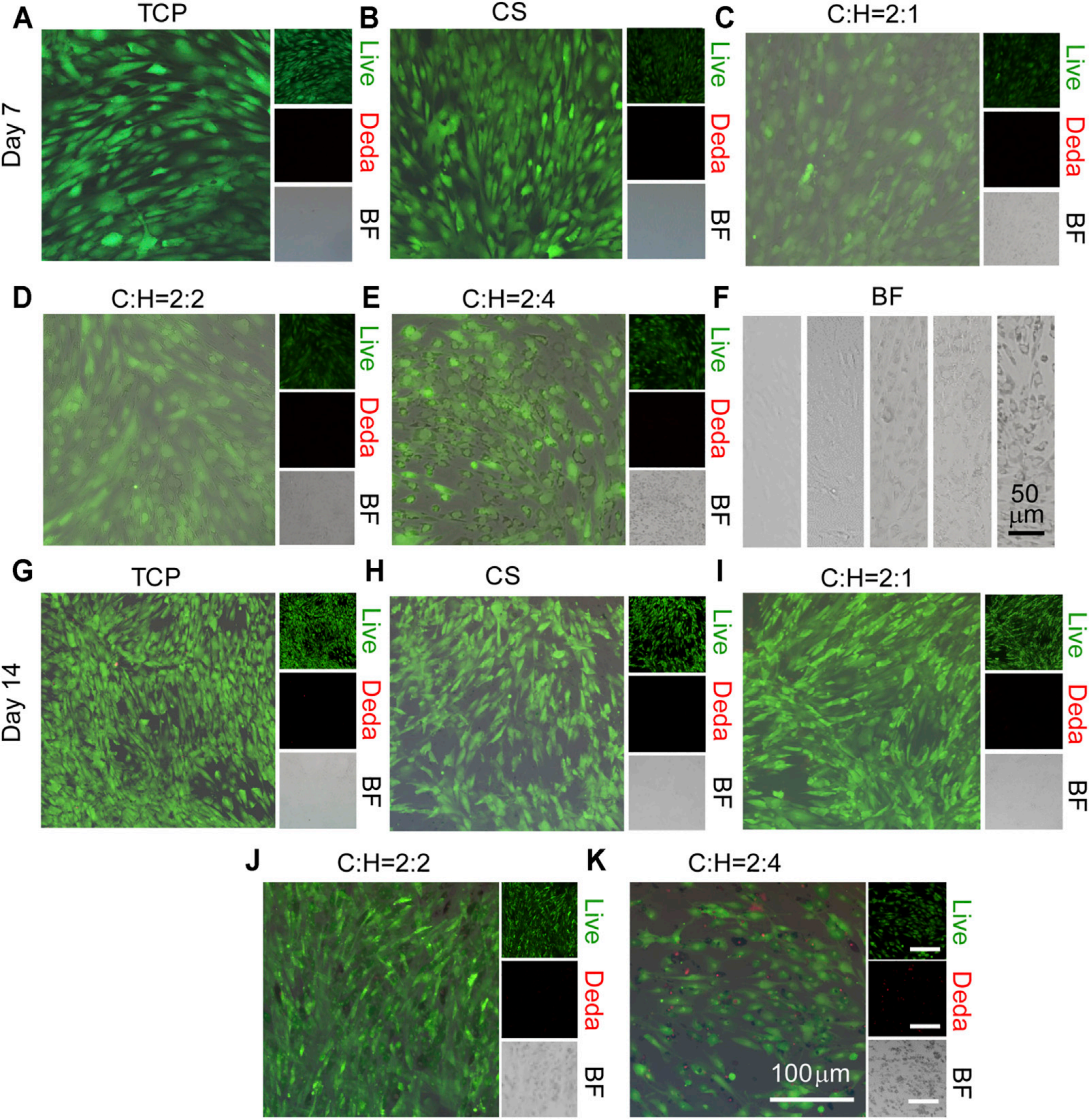
Cell viability of hADSCs cultured on the surface of the CS@HAP NWs aerogel. **(A–E)** Live and dead staining of hADSCs cultured on the surface of tissue culture plates, CS aerogels and CS@HAP NWs aerogels for 7 days and **(G–K)** 14 days in growth medium. **(F)** Bright field images of hADSCs cultured on the surface of tissue culture plates, CS aerogels and CS@HAP NWs aerogels for 7 days.

To explore the cell viability of hADSCs as influenced by the structure of the CS@HAP NWs aerogel, hADSCs were seeded on 24-well plates, and the aerogels were placed into the 24-well plates to simulate the surface of the aerogel for 7 and 14 days. As shown in Figures 5A–E, few dead cells were detected in all of the groups after 7 days. However, the living cell density in the group with a high content of HAP NWs (C:H = 2:4) aerogels decreased after 14 days of culture, as shown in Figures 5G–K.

It has been reported that the surface attachment of cells is crucial for cellular activities. Due to the degradation property of the CS in the scaffold, pieces of nanowires coated with CS break off the aerogel (Figure 5F). Thus, the surface contact signal between the matrix and the cell membrane changed significantly. Thus, cell viability and proliferation will be suppressed.

**FIGURE 6:**
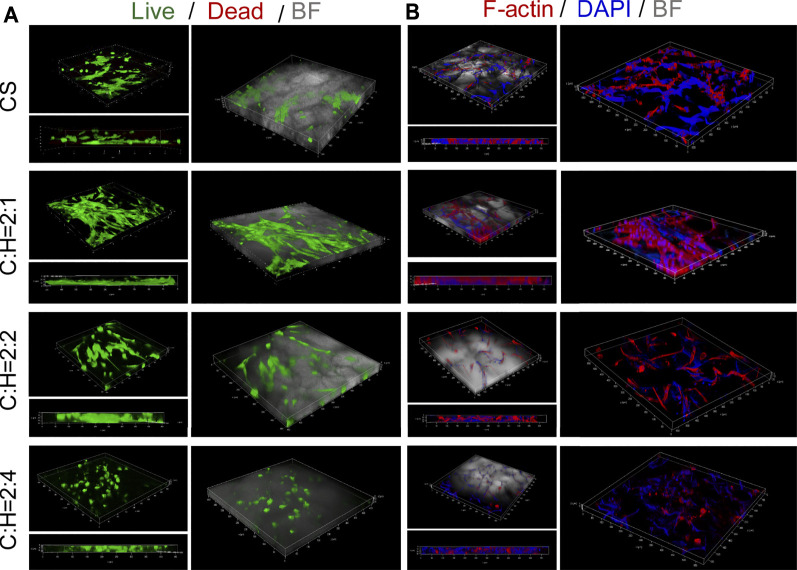
Cell viabilities and spreading morphology of hADSCs cultured within the CS@HAP NWs aerogel. **(A)** Live and dead staining of hADSCs cultured on the CS aerogel and CS@HAP NWs aerogel for 7 days. **(B)** Fluorescence microscopy images of F-actin and nuclear staining of hADSCs seeded on the CS aerogel and CS@HAP NWs aerogel for 7 days.

The cell viabilities and spreading morphology of hADSCs cultured within aerogels were observed by utilizing 3D imaging technology (Figure 6 and Supplementary Figures S1, S2). Similar to the results shown in Figure 5, few dead cells were detected in all of the groups (Figure 6A). Additionally, the cell density was low in the aerogel (C:H = 2:4). This may be due to the decreased porosity and pore size of the aerogel (Figure 2C), as well as the faster structure collapse, as discussed in Figure 5F.

Cytoskeletal and nuclear immunostaining were performed to study the cellular attachment and cell spreading morphologies. The cytoskeleton was stained red, and the nuclei were stained blue. There was no obvious difference identified in the cell morphologies of the hADSCs cultured with HAP NWs for 3 days in a 2D environment compared with the control group (Supplementary Figure S1). The cell morphology is typical of a spindle shape and spreads well. Next, we compare the adherent behavior of hADSCs cultured on 2D and 3D substrates, as illustrated in Figure 6B and Supplementary Figure S2. Most of adherent cells spread out along the pores of the 3D scaffold instead of laying on the TCP. Subject to space limitations, the cell morphology is influenced by the cavities of the aerogel scaffold. Compared with the CS aerogel group, hADSCs attached well to the walls in the CS@HAP NWs aerogel group (C:H = 2:1) and had a larger cytoskeleton spread area (Figure 6B). However, with a higher HAP content (C:H = 2:4), the cytoskeletal spread area obviously decreased. This may be attributed to the decreased porosity of the aerogel, and may also be due to the decrease in cell attachment to the wall of the cavities.

### Osteogenic Differentiation Regulation of the CS@HAP NWs Aerogel

**FIGURE 7:**
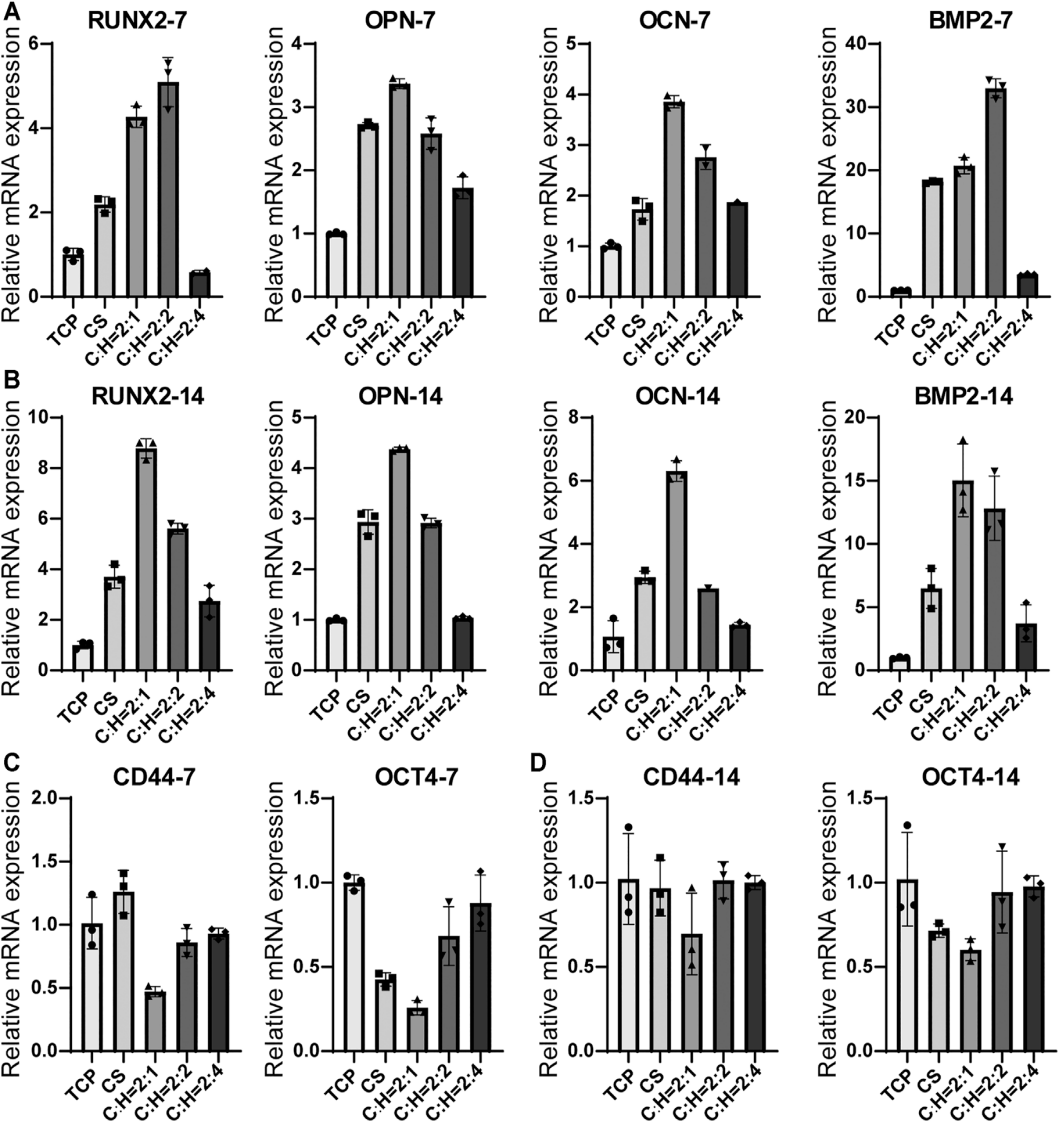
RT-qPCR analysis of osteogenic differentiation-related genes for hADSCs cultured on the tissue culturing plate, CS aerogel and CS@HAP NWs aerogel for **(A)** 7 days and **(B)** 14 days. And analysis of stemness-related genes for hADSCs cultured for **(C)** 7 days and **(D)** 14 days.

The effect of the CS@HAP NWs aerogel on the osteogenic differentiation of hADSCs can be evaluated by the expression of specific genes and proteins. The hADSCs were cultured on CS@HAP NWs aerogels with various contents of HAP NWs in differentiation medium for 7 and 14 days. The expression of osteogenesis-related genes, including RUNX2, OPN, OCN, and BMP2, was analyzed, as was the expression of stemness-related genes CD44 and OCT4 by real-time quantitative polymerase chain reaction (RT-qPCR) (Figure 7).

RUNX2 is an important transcription factor involved in bone development and maturity (Komori, 2018). After 7 days of culture (Figure 7A), the relative mRNA expression levels of RUNX2 in hADSCs cultured on the CS aerogel, C:H = 2:1 aerogel and C:H = 2:2 aerogel were upregulated by 2.2-fold, 4.3-fold and 5.1-fold, respectively, compared to those of the 2D control (TCP) group. The decreased expression of the hADSCs cultured on the C:H = 2:4 aerogel was due to the suppressed cell proliferation discussed above. Furthermore, OCN and OPN are important markers that reflect the early stage and late stage of osteogenic differentiation, respectively, (Hao et al., 2019). The gene expression levels of OPN and OCN in the cells cultured on the C:H = 2:1 aerogel increased 3.4- and 3.9-fold compared to the control. Moreover, the higher expression compared to that of the CS aerogel indicates the acceleration property of HAP NWs on the osteogenic differentiation of hADSCs in the 3D matrix. Additionally, BMP2 also plays an important role in inducing osteogenic differentiation. The expression of BMP2 reached 18.2, 20.7, and 30.0-fold in the cells cultured on C:H = 2:0, 2:1 and 2:2 aerogels compared to the control. This indicates the promotion of cell differentiation in the 3D matrix.

After 14 days of culturing (Figure 7B), the gene expression trends for all the genes did not obviously change. This confirms the solid regulatory effect of the matrix on the osteogenic differentiation of hADSCs. It should be noted that the hADSCs on the C:H = 2:1 aerogel showed the highest RUNX2 expression after a longer period of culture. So did the BMP2 expression.

OCT4 is an important transcription factor that maintains the stemness of hADSCs, and CD44 is a typical marker of hADSCs. As shown in Figure 7C, after 7 days of culture, the expression levels of OCT4 and CD44 decreased obviously in the cells cultured on the C:H = 2:1 aerogel. This result is consistent with the differentiation gene expression. This result suggests that aerogel scaffolds facilitate the differentiation of hADSCs and that the incorporation of a certain amount of HAP NWs enhances osteogenic differentiation.

**FIGURE 8:**
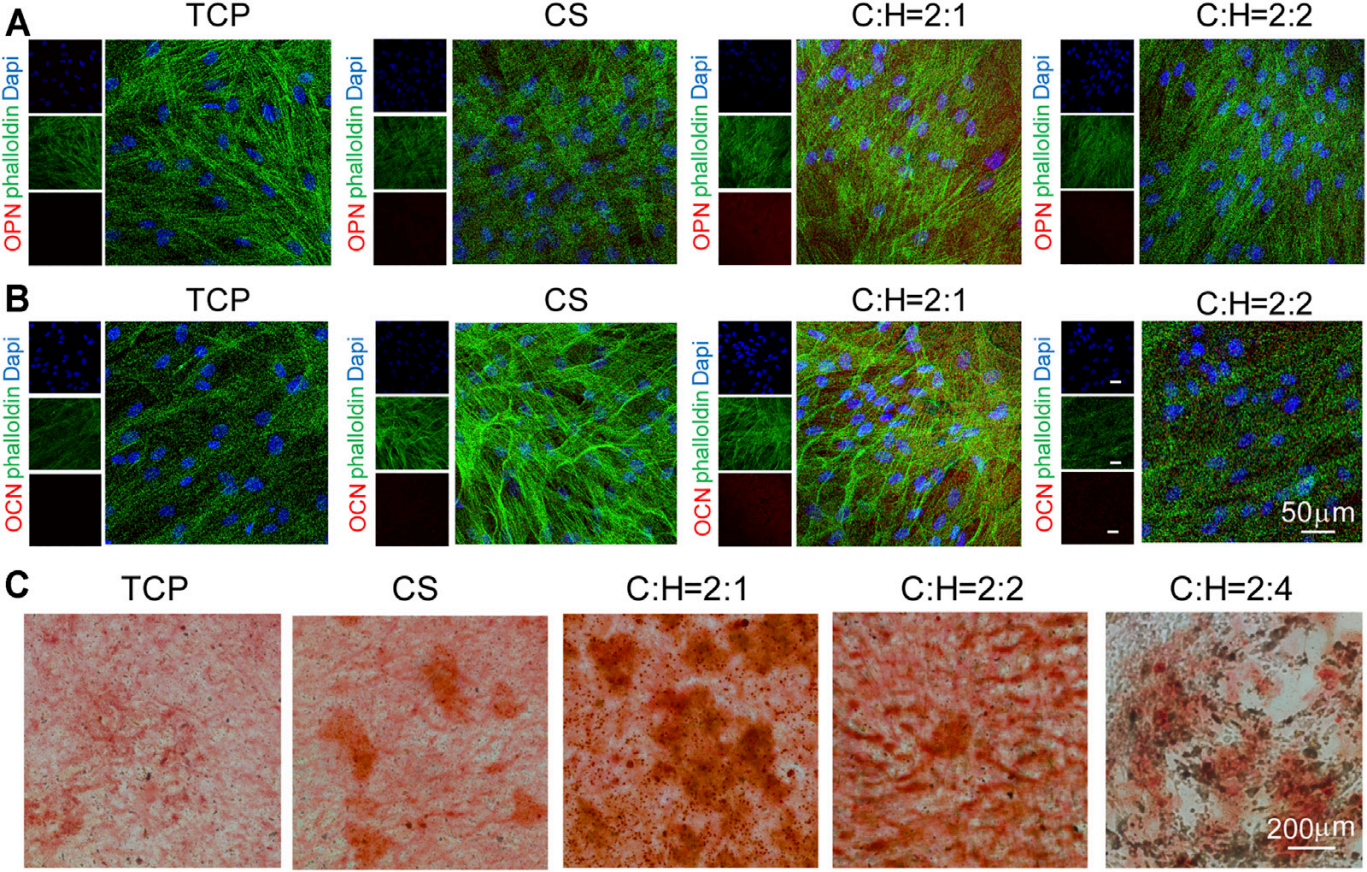
Immunofluorescence cellular staining images of osteogenic markers OPN and OCN for 14 d. **(A–B)** hADSCs were cultured on tissue culture plates, CS aerogels and CS@HAP NWs aerogels for 21 days in differentiation medium. F-actin was stained green with Alexa Fluor 488-labeled phalloidin; cell nuclei were stained blue with DAPI, and OPN and OCN were stained red. **(C)** Alizarin Red staining of hADSCs cultured on the tissue culture plate, CS aerogel and CS@HAP NWs aerogel for 21 days in differentiation medium.

The expression of some specific osteogenic proteins was studied by immunofluorescence staining (Figure 8). After culturing hADSCs on 2D plates and 3D aerogels for 14 days, the expression of osteogenesis-related proteins OPN and OCN was analyzed (Figures 8A,B). The fluorescence intensity of OPN and OCN in the cells cultured on the C:H = 2:1 aerogel group was the highest compared to that of the other aerogel groups. This result is consistent with the RT-qPCR result, suggesting the potent effect of the CS@HAP NWs aerogel in promoting osteogenic differentiation. The formation of mineralized nodules was another crucial marker of osteogenic differentiation. As shown in Figure 8C, Alizarin Red staining was performed to evaluate the efficiency of calcium deposition. After 21 days of culture, more red calcium modules were found in cells cultured on the C:H = 2:1 aerogel group, indicating the strong osteogenic ability of the C:H = 2:1 aerogel, which is consistent with the RT-qPCR and immunostaining results.

## Conclusion

In summary, we established highly porous 3-dimensional (3D) chitosan aerogel scaffolds with the incorporation of various amounts of hydroxyapatite nanowires (HAP NWs) by a freeze-drying method. The structure of the CS@HAP NWs aerogel was tailored to simulate cancellous bone, which was confirmed by the interconnected porous structure of the CS@HAP NWs aerogel. High porosity and proper mechanical properties can effectively benefit the osteogenic differentiation of human adipose-derived stem cells (hADSCs). This work not only regulated the osteogenic properties of hADSCs by engineering hydroxyapatite 3D-hybrid scaffolds with cancellous bone structures but also provides a deep understanding of the improved cellular activity of stem cells growing in 3D aerogel scaffolds. Therefore, this work can be applied to tailor 3D biomimetic scaffolds with proper physical and biochemical properties for bone tissue engineering.

## Data Availability

The original contributions presented in the study are included in the article/Supplementary Material, further inquiries can be directed to the corresponding authors.
