# Supplementary material for: Osteogenic Property Regulation of Stem Cells by a Hydroxyapatite 3D-Hybrid Scaffold With Cancellous Bone Structure
Source: Front Chem. 2021 Nov 19;9:798299. doi: 10.3389/fchem.2021.798299 (PMC8640089; doi:10.3389/fchem.2021.798299)
Supplement: Supplementary file 1 [file DataSheet1.docx]

Supplementary Materials of

Osteogenic property regulation of stem cells by a hydroxyapatite 3D-hybrid scaffold with cancellous bone structure

He Xia^1†^, Lun Dong^2†^, Min Hao^1^, Yuan Wei^3^, Jiazhi Duan^1^, Xin Chen^1^, Liyang Yu^1^, Haijun Li^4*^, Yuanhua Sang^1*^, Hong Liu^1^

^1^State Key Laboratory of Crystal Materials, Shandong University, Jinan 250100, P. R. China

^2^Department of Breast Surgery, Qilu Hospital, Shandong University, Jinan 250012, China

^3^Department of Obstetrics and Gynecology, Qilu Hospital, Shandong University, Jinan 250012, China

^4^Key Laboratory of Cardiovascular Proteomics of Shandong Province, Department of Geriatric Medicine, Qilu Hospital, Shandong University, Jinan 250012, China

*** Correspondence:**

Corresponding Authors

[sangyh@sdu.edu.cn](mailto:sangyh@sdu.edu.cn) (Y. Sang); [187307923@qq.com](mailto:187307923@qq.com) (H. Li)


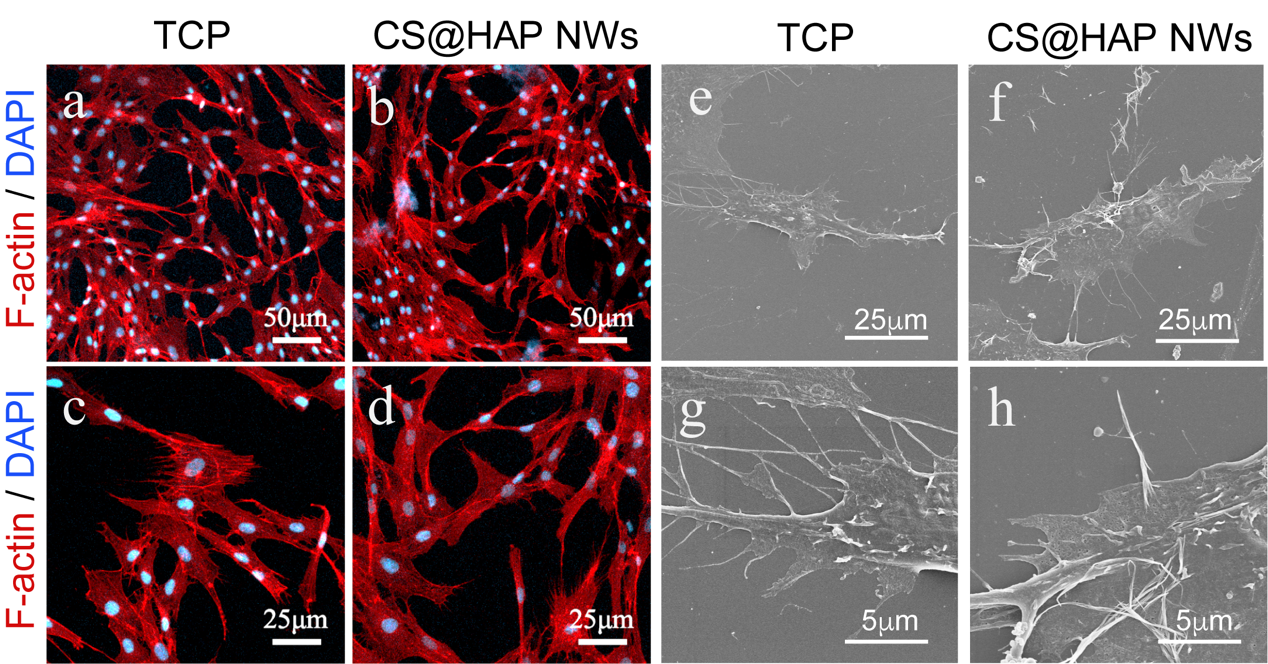


**Supplementary Figure 1.** The spreading and morphologies of hADSCs culturing with 2D HAP nanowires. (a-d) Fluorescence microscopy images of F-actin and nuclei staining of hADSCs culturing with 0 and 100 μg/ml HAP nanowires for 3 days in growth meidum. (e-h) SEM images of hADSCs culturing with 0 and 100 μg/ml HAP nanowires for 3 days in growth meidum.

As shown in Fig S1 a-d, hADSCs culturing with 2D HAP nanowires for 3 days showed a spindle shape which is similar with the morphology of hADSCs cultured on TCP group. The results showed that the morphology of stem cells was not affected by the addition of HAP nanowires. SEM images were shown in Fig S1 e-h and the surface of hADSCs were observed. With the addition of HAP nanowires, we can find some nanowires stick on the cell membrane and the cells have more filopodia than the control which demonstrate the nanowires would not affect cell migration and adhesion.

**
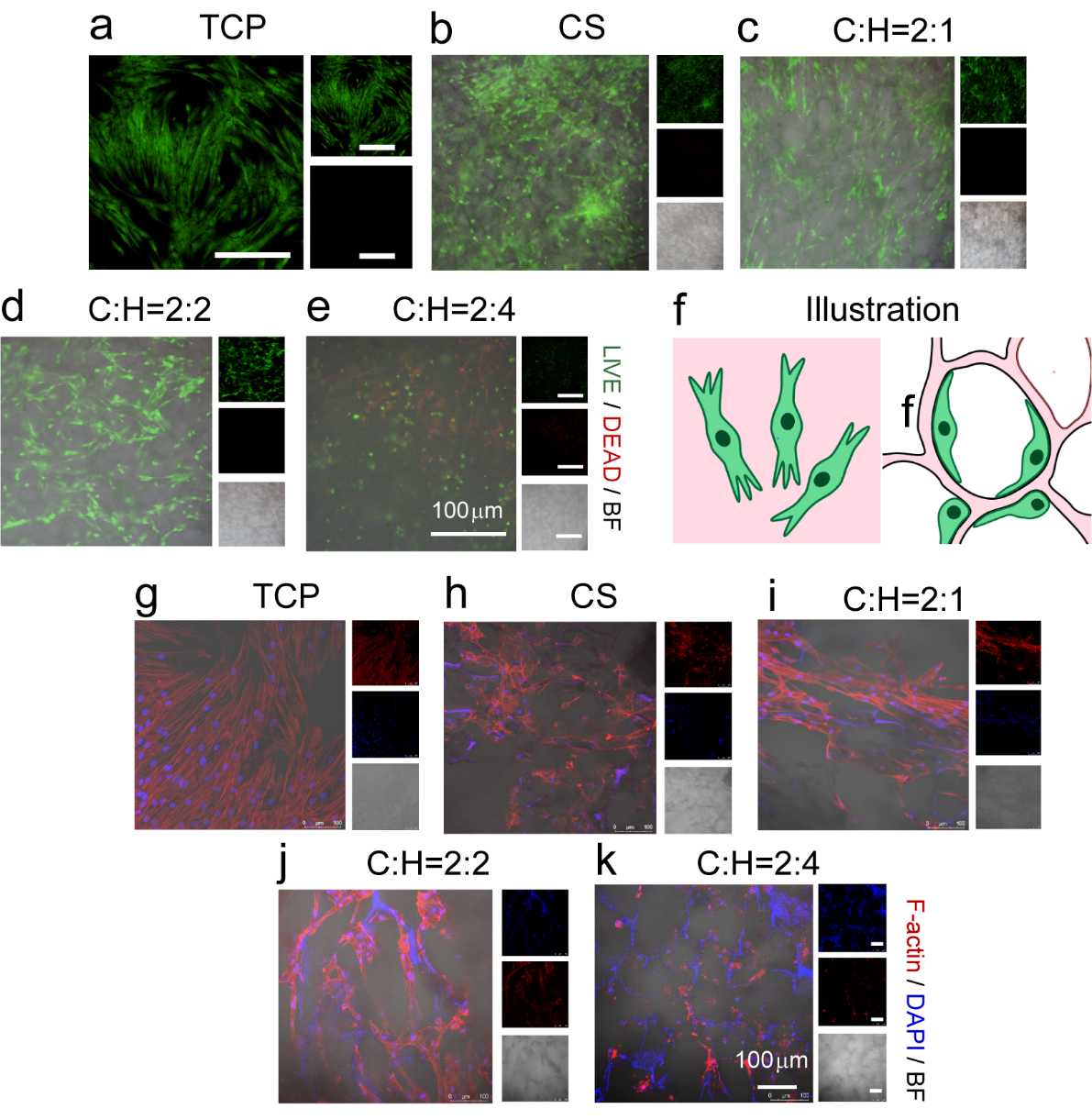
**

**Supplementary Figure 2.** Cell viabilities and spreading morphology of hADSCs culturing with CS@HAP aerogel in 3D environment. (a) Live and dead staining of hADSCs culturing on CS aerogel and CS@HAP aerogel for 7 days captured by Inverted fluorecent microscope. (b) 3D stack images of F-actin and nuclei staining of hADSCs seeded into CS aerogel and CS@HAP aerogel for 7 days captured by Confocal microscope.

As shown in Fig S2, hADSCs lived and spread well on CS, C:H=2:1 and C:H=2:2 aerogels while the cells turned round in C:H=2:4 group. The results were consistent with the mechanical properties of CS@HAP NWs aerogel that the porosity and pore size was secreased in C:H=2:4 group. We also compare the adherent behavior of hADSCs cultured on 2D and 3D substratate, as illustration shown in Fig S2. Most of adherent cells spread out along the pores of the scaffold rather than as individual spheres of cells, which suggests that the volum of pore, porosity and other properties of the scaffold are very important to direct the fate of stem cells.

**Supplementary Table S1.** Sequences of RT–qPCR Primers.

| Gene | Forward primers (5’-3’) | Reverse primers (5’-3’) |
| --- | --- | --- |
| RUNX2 | ACGAATGCACTATCCAGCCA | GCAGGTACGTGTGGTAGTGA |
| OPN | CCTCCTAGGCATCACCTGTG | CCACACTATCACCTCGGCC |
| OCN | CTGTATCAATGGCTGGGAGG | GCCTGGAGAGGAGCAGAACT |
| BMP2 | ACCCTTTGTACGTGGACTTC | GTGGAGTTCAGATGATCAGC |
| CD44 | TCAGGAGATATGCAAAGCAGAA | TTGCCTCTCACTCGGTTCTC |
| OCT4 | TCAGCCAAACGACCATCTGC | AGCCTGGGGTACCAAAATGG |
| β-actin | CATGTACGTTGCTATCCAGGC | CTCCTTAATGTCACGCACGAT |
